# In Situ-Grown MIL-100(Fe) for Interfacial Regulation of KTBC and Its Adsorption Performance and Mechanism for Xylenol Orange Removal

**DOI:** 10.3390/molecules31152604

**Published:** 2026-07-25

**Authors:** Shirui Zheng, Jinting Jiang, Zhihao Fang, Fangfang Liu, Yongwei Li

**Affiliations:** 1 State Key Laboratory of Bio-Fibers and Eco-Textiles, College of Materials Science and Engineering, Shandong Collaborative Innovation Center of Marine Biobased Fibers and Ecological Textiles, Qingdao University, Qingdao 266071, China; threezheng@aliyun.com; 2 School of Chemical Engineering and Environment, Weifang University of Science and Technology, Weifang 262700, China; jinn0717@163.com (J.J.); wfsgfzh@163.com (Z.F.); liuff10507@wfust.edu.cn (F.L.)

**Keywords:** MIL-100(Fe)@KTBC, dye wastewater, adsorption, mechanism

## Abstract

In this study, a MIL-100(Fe)@KTBC composite was successfully fabricated via an in situ hydrothermal method using KOH-activated tomato-biochar-derived carbon (KTBC) as the support, and was applied for the efficient adsorptive removal of xylenol orange (XO) from water. Characterization by SEM, XRD, FTIR, XPS, and BET confirmed that MIL-100(Fe) was successfully loaded onto the KTBC surface, and the resulting composite exhibited a well-developed porous structure, abundant functional groups, and good thermal stability. Adsorption experiments showed that MIL-100(Fe)_0.5_@KTBC delivered the optimal performance, with a maximum adsorption capacity of 246.12 mg/g; high removal efficiency was achieved at pH 4.0 and an adsorbent dosage of 0.4 g/L. The adsorption process followed pseudo-second-order kinetics and the Langmuir isotherm model, indicating spontaneous, endothermic, monolayer adsorption dominated by chemisorption. The composite also demonstrated strong resistance to interfering ions and favorable reusability. This work provides a scientific basis for the development of efficient and stable biochar-based MOF composites for the treatment of printing and dyeing wastewater.

## 1. Introduction

Dyeing wastewater is one of the major sources of industrial pollution and is extensively generated in industries such as textiles, papermaking, pharmaceuticals, and leather manufacturing [1,2]. Synthetic dyes in wastewater commonly contain complex aromatic chromophoric groups, including azo [3], anthraquinone [4], and arylmethane [5] moieties, and are characterized by high toxicity, resistance to photolysis and oxidation, poor biodegradability, and environmental persistence [6]. These pollutants and their degradation products not only pose serious health risks to humans [7], including carcinogenicity, mutagenicity, and organ damage [8], but also disrupt the balance of aquatic ecosystems [9] and amplify toxicity through bioaccumulation along the food chain [10]. Therefore, scientifically sound and effective treatment measures must be implemented prior to discharge to reduce environmental risks and health hazards [11].

Existing dye wastewater treatment technologies include physical methods [12], such as membrane separation, coagulation, and adsorption; chemical methods [13], such as oxidation, chemical precipitation, and ion exchange; and biological methods [14]. However, these approaches generally suffer from limited efficiency, high cost, and secondary pollution [15,16]. Among them, adsorption has become a research hotspot owing to its operational simplicity, high efficiency, low cost, and the regenerability of adsorbents [17,18]. Biochar (BC), as an environmentally friendly adsorbent [19], features abundant feedstock availability, low cost, and well-developed porous structures [20,21]. Nevertheless, its adsorption performance remains constrained by its surface chemistry and pore structure [22]. Metal–organic frameworks (MOFs) have demonstrated excellent adsorption potential because of their ultrahigh specific surface areas and tunable porosity [23,24]. In particular, MIL-100(Fe), which is rich in Fe^3+^ active sites [25] and functional groups such as carboxyl groups [26], can efficiently adsorb anionic dyes through mechanisms including electrostatic attraction, complexation, and π–π stacking [27]. However, MIL-100(Fe) suffers from several drawbacks, including particle aggregation, difficult separation, high cost, and insufficient water stability, which limit its engineering applications [28].

Combining MOFs with biochar enables complementary advantages [29]. As an ideal support, biochar can not only disperse MOF particles through in situ growth and inhibit their aggregation [30], but also synergistically enhance adsorption performance by virtue of its intrinsic pore structure [31]. In addition, its low cost and favorable mechanical stability help address the separation challenges of MOFs and improve their recyclability [32]. More importantly, the two components exhibit pronounced structure–performance synergy [33]: the hierarchical micro–meso–macroporous architecture of biochar provides interconnected diffusion channels, thereby facilitating mass transfer and improving the utilization efficiency of MOF active sites [34]; the porous carbon framework restricts MOF aggregation and promotes the uniform dispersion of crystals, exposing more coordination sites [35]; meanwhile, the mechanical elasticity of biochar can buffer stress, preserve MOF crystallinity, and significantly enhance the cycling stability and regeneration performance of the composites [36]. Therefore, the development of biochar/MOF composites represents an important strategy for overcoming the limitations of individual materials and achieving efficient treatment of dye-containing wastewater [37]. Recent studies have demonstrated that the in situ growth of MOF on biochar is an effective strategy for improving dye removal [29]. Fan and Zhang fabricated a CSB/PDA/ZIF-8 composite by growing ZIF-8 in situ on polydopamine-modified coconut-shell biochar [38]. The composite showed maximum adsorption capacities of 1568 and 1496 mg/g for malachite green and rhodamine B, respectively, owing to pore filling, electrostatic attraction, hydrogen bonding, and π–π interactions. Similarly, UiO-66 and UiO-67 were solvothermally grown on poplar-leaf biochar for Congo red removal [31]; UiO-67@BY exhibited a Langmuir capacity of 940.70 mg/g.

Xylenol Orange, a triphenylmethane derivative, possesses an extensively conjugated π-electron system that confers exceptional photothermal and biochemical stability. The presence of phenolic hydroxyl and sulfonic acid groups renders XO anionic and highly water-soluble, characteristics that impede its effective removal by conventional water treatment processes [39]. As a potent metal chelating agent, XO discharged into the environment is not only resistant to degradation but also forms stable complexes with heavy metals. This complexation enhances the recalcitrance of XO while simultaneously facilitating the migration and bioaccumulation of heavy metals, thereby inducing synergistic ecotoxicological effects. In light of these concerns, this study selected XO as a model pollutant to systematically evaluate the efficacy of adsorbent materials in treating dye-laden wastewater.

Tomato stalks were selected as the biochar precursor because they are abundant in quantity and concentrated in source, and are usually regarded as low-value agricultural waste. Their conversion into functional carbon materials therefore provides both environmental and economic benefits. Compared with conventional wood- or crop-straw-derived biochars, tomato stalks biochar contains a carbonaceous framework originating from a biomass rich in cellulose, hemicellulose, lignin, and naturally occurring oxygen-containing components. After carbonization and KOH activation, this framework can develop a highly porous structure, enlarged specific surface area, abundant defect sites, and surface oxygen-containing functional groups. These characteristics are advantageous for the heterogeneous nucleation and immobilization of MIL-100(Fe) because they facilitate precursor adsorption, improve interfacial interactions between the carbon support and Fe-based nodes, and reduce the aggregation of MOF particles during in situ growth.

The originality of this study therefore lies in the coupled valorization and functionalization strategy: tomato-processing waste is transformed into a KOH-activated hierarchical carbon support, followed by the in situ construction of MIL-100(Fe) on its surface and within its accessible pore network. The resulting composite is expected to integrate the adsorption capacity, conductivity, and structural robustness of activated biochar with the high density of Fe-based active sites and tunable porosity of MIL-100(Fe). This synergistic architecture provides improved active-site exposure, interfacial contact, mass-transfer efficiency, and resistance to particle agglomeration, offering a distinct design route for high-performance biomass-derived MOF composites.

In this study, high-specific-surface-area KTBC was used as a support, and MIL-100(Fe)@KTBC composites were prepared via an in situ hydrothermal method for the adsorptive removal of XO from simulated wastewater. Batch adsorption experiments, combined with adsorption kinetic, isotherm, and thermodynamic analyses, were conducted to elucidate the adsorption behavior and underlying mechanisms of the composites. Regeneration and cycling stability were evaluated through cyclic regeneration experiments, and the adsorption mechanism was further investigated to verify the practical application potential of the material.

## 2. Results and Discussion

### 2.1. Characterization of the Adsorbents

**Figure 1:**
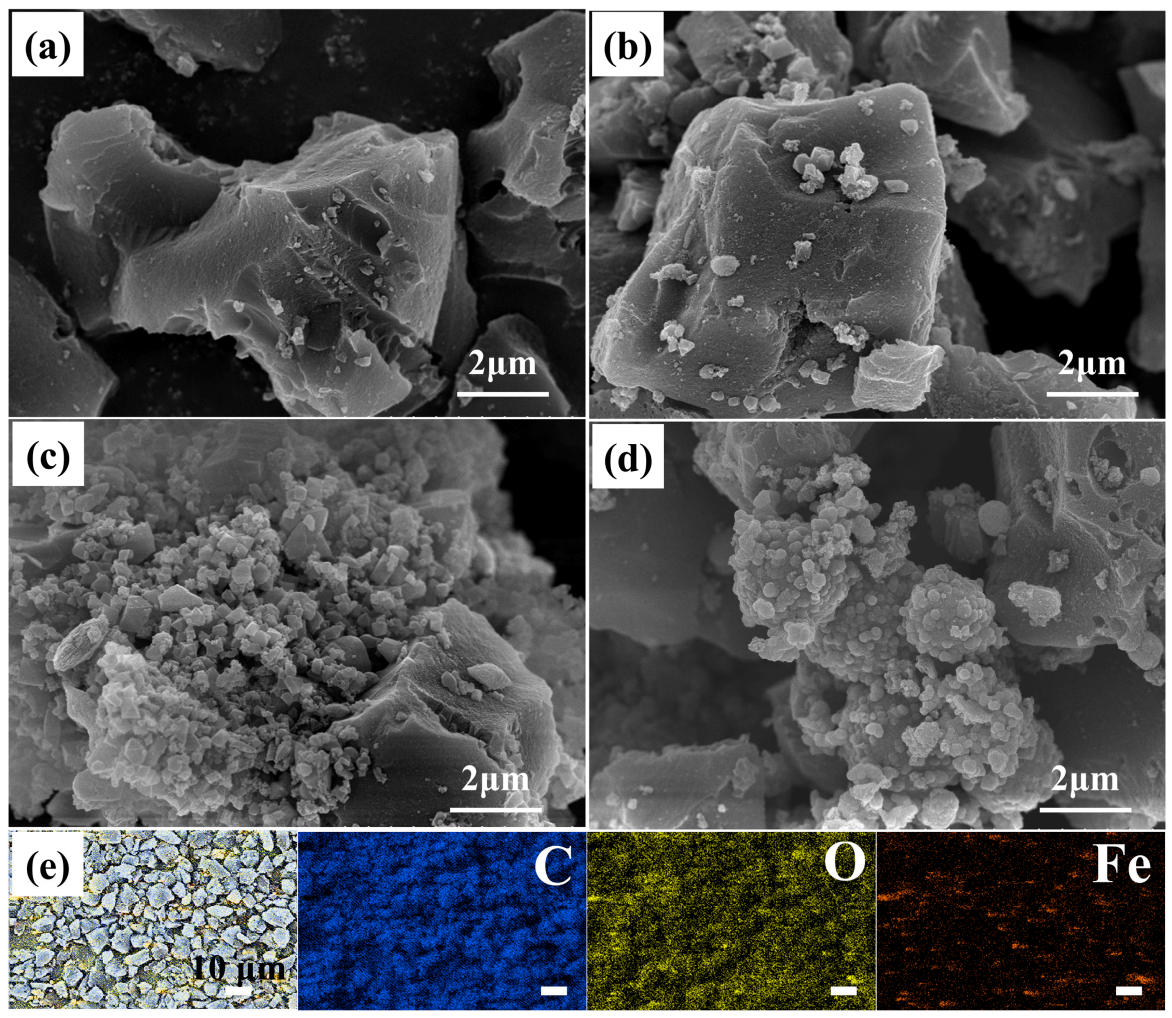
SEM images of (**a**) KTBC, (**b**) MIL-100(Fe)_0.3_@KTBC, (**c**) MIL-100(Fe)_0.5_@KTBC, (**d**) MIL-100(Fe)_0.7_@KTBC, (**e**) TEM image of MIL-100(Fe)_0.5_@KTBC.

As shown in Figure 1, with the increase of MIL-100(Fe) loading from 0.3 to 0.7, the surface morphology of the composite gradually transformed from relatively smooth KTBC lamellae to a rough particle-loaded structure, indicating that MIL-100(Fe) was successfully grown in situ and anchored onto the KTBC surface. Among these composites, MIL-100(Fe)_0.5_@KTBC exhibited relatively uniform particle dispersion combined with a high loading amount, whereas MIL-100(Fe)_0.7_@KTBC displayed obvious agglomeration, which is detrimental to the full exposure of active sites and the mass transfer process. The above morphological analysis demonstrates that a loading ratio of 0.5 is more favorable for the formation of a composite with homogeneous dispersion and intimate interfacial bonding [40]. Furthermore, the SEM-EDS mapping results of MIL-100(Fe)_0.5_@KTBC in Figure 1e reveal that C, O, and Fe elements are continuously and uniformly distributed on the material surface, further confirming that MIL-100(Fe) has been homogeneously loaded in situ on the KTBC surface [37].

**Figure 2:**
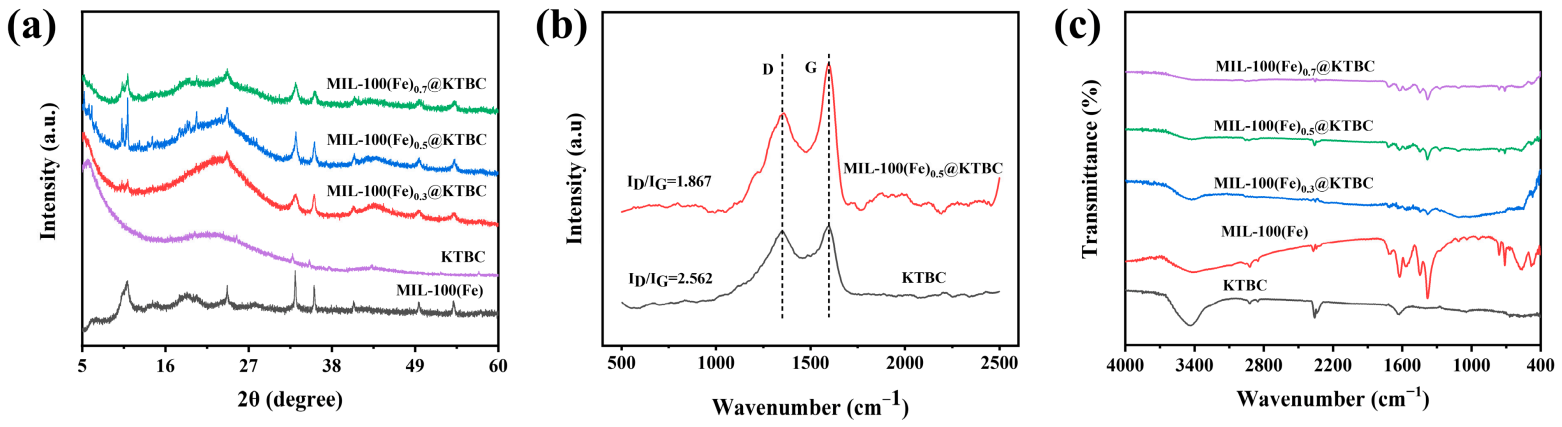
(**a**) XRD patterns, (**b**) Raman spectra, and (**c**) FTIR spectra.

XRD analysis (Figure 2a) shows that KTBC exhibits only a broad, weak diffraction peak at 2θ = 21.7° with no sharp characteristic peaks, indicating an amorphous carbon structure [41]. In contrast, MIL-100(Fe) displays clear characteristic diffraction peaks at 2θ = 10.26°, 10.98°, 24.21°, 33.21°, 35.68°, among others. These peaks confirm that the crystalline structure of MIL-100(Fe) was well preserved [42]. The XRD patterns of the MIL-100(Fe)_x_@KTBC composites all retain the characteristic peaks of MIL-100(Fe). Furthermore, their intensities gradually increase as x increases from 0.3 to 0.7. These results demonstrate that MIL-100(Fe) was successfully deposited on the biochar surface while retaining its original crystalline phase [34]. Moreover, the biochar substrate endows the MOF with favorable dispersion and structural stability without significantly affecting its crystal phase.

As shown in Figure 2b, both KTBC and the MIL-100(Fe)_0.5_@KTBC composite exhibited characteristic D and G bands of carbon materials at 1359 cm^−1^ and 1592 cm^−1^, respectively [37]. The I_D_/I_G_ ratio of KTBC was 2.562, indicating abundant defects and a low degree of graphitization; after compositing, the I_D_/I_G_ ratio decreased to 1.867, suggesting that the in situ growth of MIL-100(Fe) contributed to the repair of partial defects and the improvement of carbon structural order. The composite still retained distinct D and G bands, indicating that the MOF loading did not disrupt the carbon skeleton. No sharp characteristic peaks of MIL-100(Fe) were observed, which may be attributed to the masking of ligand and Fe–O vibrations by the strong carbon signals of biochar [37]. The adsorption between defect sites and oxygen-containing functional groups was weakened by the decrease in carbon defect content; however, the π–π interactions between the aromatic carbons of KTBC and the aromatic rings of XO were enhanced by the regular and continuous sp^2^-conjugated carbon skeleton. Carbon defects originally filled with MIL-100(Fe) were transformed into stable MOF–carbon heterointerfaces, and abundant Fe^3+^ active binding sites were generated simultaneously. In summary, MIL-100(Fe) was successfully composited with KTBC, and the biochar structure was modulated through interfacial interactions.

As shown in Figure 2c, KTBC displayed characteristic peaks at 3434.6, 2921.6, 2852.2, 1627.6, and 1052.9 cm^−1^, which were attributed to O–H stretching, C–H stretching, and C=O stretching vibrations, respectively [41]. For MIL-100(Fe), the absorption bands at 476.3 and 568.9 cm^−1^ corresponded to Fe–O and Fe–O–Fe stretching vibrations [43]; the peaks at 711.6 and 763.6 cm^−1^ originated from aromatic C–H vibrations of the trimesic acid ligand; the band around 1380.7 cm^−1^ was assigned to carboxyl C–O stretching; and the peaks at 1569.7 and 1623.7 cm^−1^ were associated with C=C and C=O stretching vibrations, respectively. The weak peaks at 1710.5 and 1448.2 cm^−1^ indicated the presence of a small amount of unreacted trimesic acid [44]. In the MIL-100(Fe)_x_@KTBC composites, with increasing MIL-100(Fe) loading, the O–H peak around 3400 cm^−1^ gradually weakened [45]; the peak profile near 1600 cm^−1^ progressively approached that of MIL-100(Fe), and the characteristic peaks in the 1000–400 cm^−1^ region intensified, confirming the successful synthesis of the composites [37].

**Figure 3:**
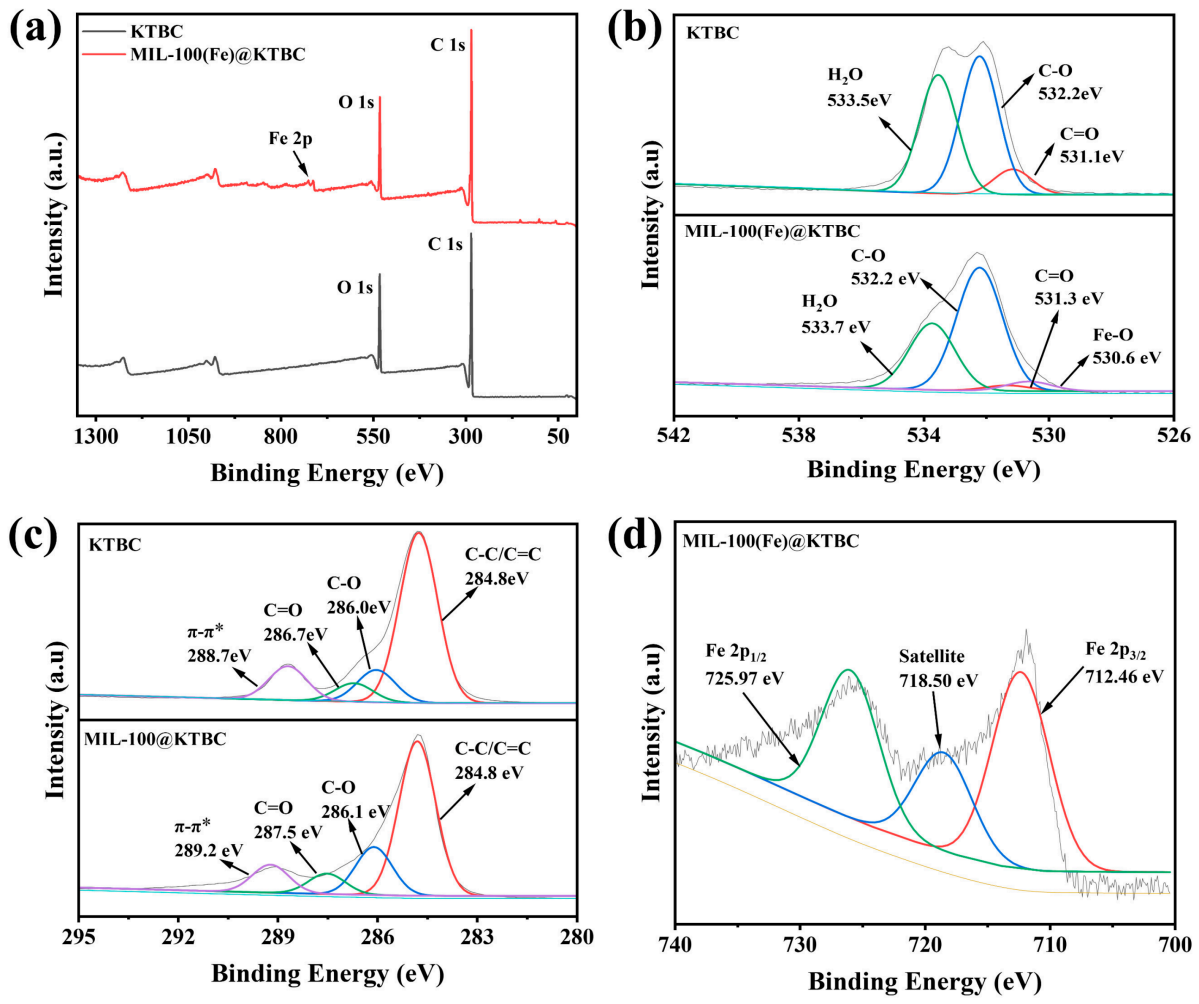
XPS spectra of KTBC and MIL-100(Fe)@KTBC composite: (**a**) survey spectrum, (**b**–**d**) high-resolution spectra of O 1s, C 1s, and Fe 2p.

As shown in Figure 3a, the XPS survey spectrum of KTBC contains only C 1s and O 1s peaks, indicating that the surface is mainly composed of carbon and oxygen. In contrast, MIL-100(Fe)@KTBC shows an additional Fe 2p signal, confirming the successful introduction of iron and preliminarily demonstrating the successful construction of the composite material. The chemical states of the elements were further analyzed using high-resolution XPS spectra. In the O 1s spectrum (Figure 3b), KTBC can be deconvoluted into three peaks corresponding to C=O at 531.2 eV, C–O at 532.2 eV, and adsorbed water/surface hydroxyl groups at 533.5 eV [36]. For MIL-100(Fe)@KTBC, four peaks are observed at 530.6, 531.3, 532.2, and 533.7 eV, which are assigned to Fe–O, C=O, C–O, and H_2_O [35], respectively. The newly emerged Fe–O peak confirms the formation of iron–oxygen coordination structures. The C 1s spectrum (Figure 3c) of KTBC shows peaks at 284.8, 286.0, 286.7, and 288.7 eV, which are attributed to C=C/C–C, C–O, C=O, and π–π* shake-up satellite, respectively [36]. After compositing, the corresponding peaks shift to 284.8, 286.1, 287.5, and 289.2 eV. The shifts in the C–O, C=O, and π–π* satellite peaks indicate chemical interactions between MIL-100(Fe) and the oxygen-containing functional groups on the KTBC surface, rather than simple physical mixing [35]. The Fe 2p spectrum (Figure 3d) displays Fe 2p3/2 at 712.46 eV, Fe 2p1/2 at 725.97 eV, and a satellite peak at 718.50 eV, all characteristic of Fe^3+^ [43], suggesting that the iron valence state remains unchanged and that the framework structure is stable. Overall, the appearance of the Fe 2p peak in the survey spectrum, the Fe–O bond in the O 1s spectrum, the shifts in the C 1s peaks, and the Fe^3+^ signals collectively confirm that MIL-100(Fe) was successfully loaded onto the KTBC surface while preserving the iron-based MOF structure.

**Figure 4:**
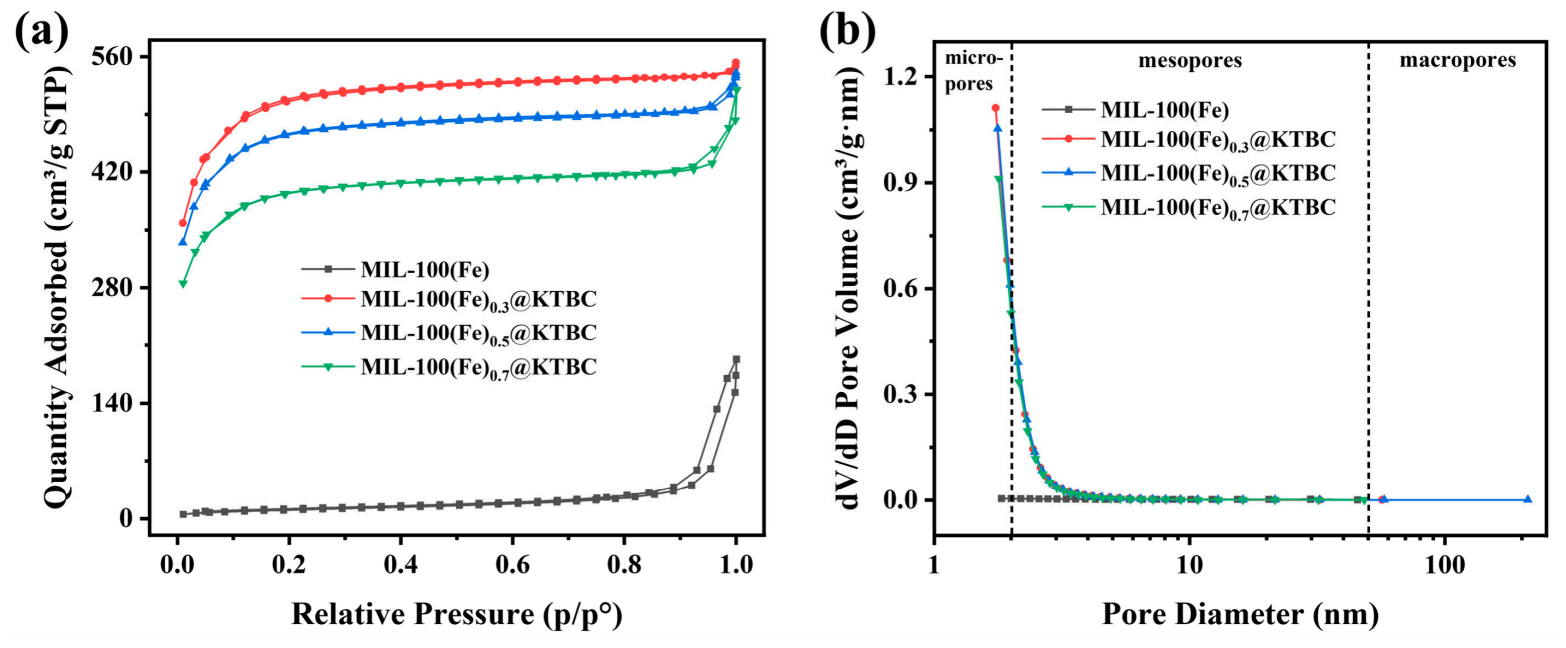
(**a**) Nitrogen adsorption–desorption isotherms and (**b**) pore size distribution curves of MIL-100(Fe) and its composites.

Figure 4 presents the N_2_ adsorption–desorption isotherms and pore size distribution curves of MIL-100(Fe) and the various MIL-100(Fe)@KTBC composites. According to the IUPAC classification, both MIL-100(Fe) and the MIL-100(Fe)@KTBC composites exhibit type IV isotherms with H3-type hysteresis loops [45]. The pore size distribution curves show that the composites contain micropores, mesopores, and macropores, with most pore sizes falling within the range of 2–20 nm. These results indicate that the composites are predominantly mesoporous materials, with mesopore sizes ranging from 2 to 50 nm. They also contain a certain proportion of micropores smaller than 2 nm, suggesting that micropores and mesopores make the dominant contributions to the overall pore structure [46]. The incorporation of MIL-100(Fe) into KTBC significantly promoted pore development and enhanced the N_2_ adsorption capacity of the resulting composites. Therefore, an appropriate MIL-100(Fe) loading is essential for constructing MIL-100(Fe)@KTBC composites with a well-developed and efficient porous structure. In contrast, excessive MIL-100(Fe) loading may compromise these structural advantages by causing pore blockage and particle aggregation.

The specific surface area and pore structure data of the different MIL-100(Fe)@KTBC samples were presented in Table 1. The specific surface area of the MIL-100(Fe)@KTBC composite initially increased and then decreased as the MIL-100(Fe) ratio was increased. At a low MIL-100(Fe) ratio, the MOF nanoparticles were uniformly dispersed, and the specific surface area was synergistically enhanced in combination with the KTBC biochar. As the MIL-100(Fe) ratio was further increased, excessive crystal growth and mutual adhesion were induced by the excess MOF precursor. Consequently, large agglomerates were formed, which covered or blocked the pore openings and internal channels of the biochar.

**Figure 5:**
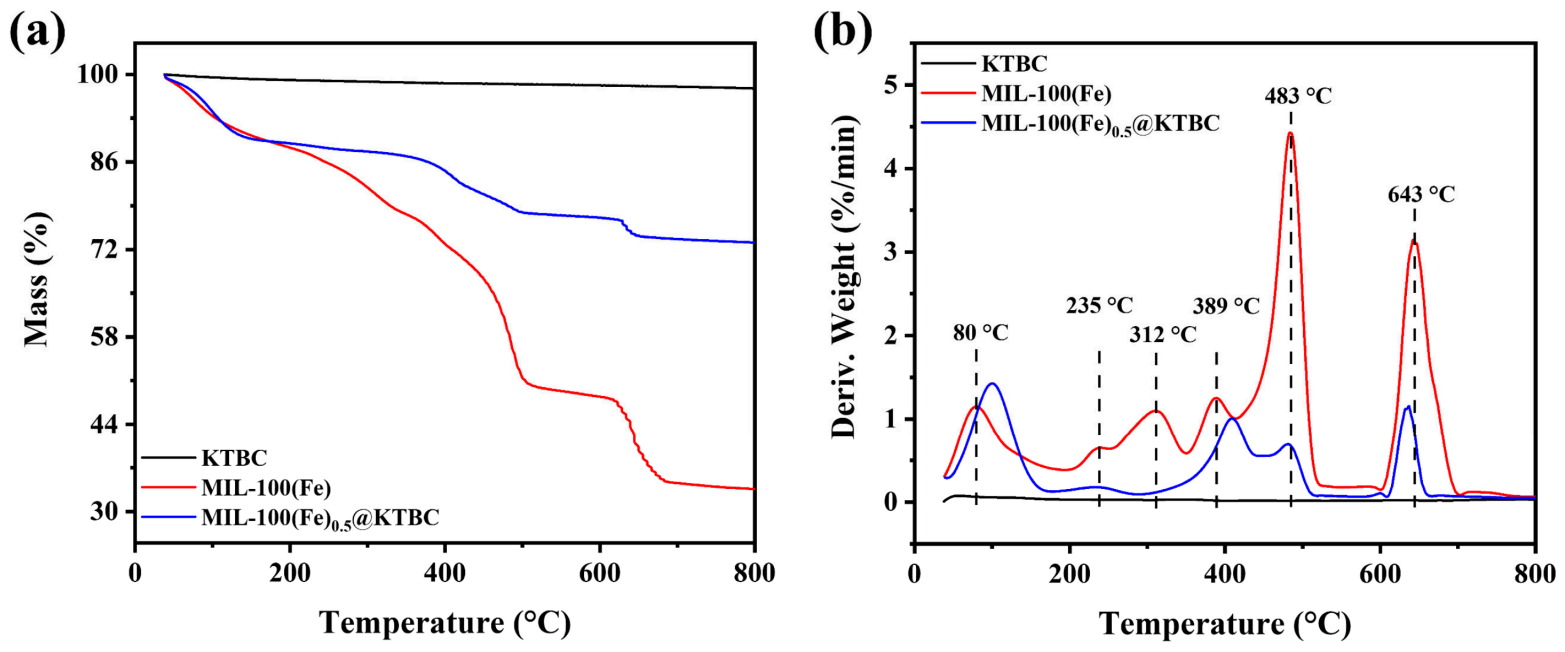
Thermogravimetric analysis of KTBC, MIL-100(Fe) and MIL-100(Fe)_0.5_@KTBC: (**a**) TG curves; (**b**) DTG curves.

The TG and DTG curves in Figure 5 show that KTBC loses only 2.25% of its mass from 0 to 800 °C, demonstrating excellent thermal stability and suitability as a stable support. MIL-100(Fe) exhibits an 11.73% weight loss below 200 °C, attributed primarily to the removal of adsorbed water and residual solvents [44], which corresponds to a weak DTG peak at 80 °C. A weight loss of 20.98% is observed between 200 and 450 °C, corresponding to the gradual decomposition of organic ligands and the initial collapse of the framework, with multiple decomposition peaks at 235, 312, and 389 °C [42]. The main weight loss stage occurs at 450–695 °C (32.68%), indicating complete destruction of the MOF framework and the formation of metal oxide residues; the strong peak at 483 °C is associated with extensive ligand decomposition and violent framework collapse [42], and above 695 °C, metal oxide residues remain. In contrast, MIL-100(Fe)_0.5_@KTBC undergoes only a slow weight loss of 2.77% over 150–350 °C and a loss of 12.86% over 350–655 °C, demonstrating that the introduction of KTBC effectively mitigates the rapid thermal decomposition of the MOF structure. The final residue of the composite consists mainly of metal oxides and a stable carbon framework [45], indicating that KTBC retards MOF decomposition through physical support and thermal protection, reflecting a synergistic thermal stabilization effect.

### 2.2. Adsorption Investigation

#### 2.2.1. Effect of Raw Material Ratio on Adsorption Performance

**Figure 6:**
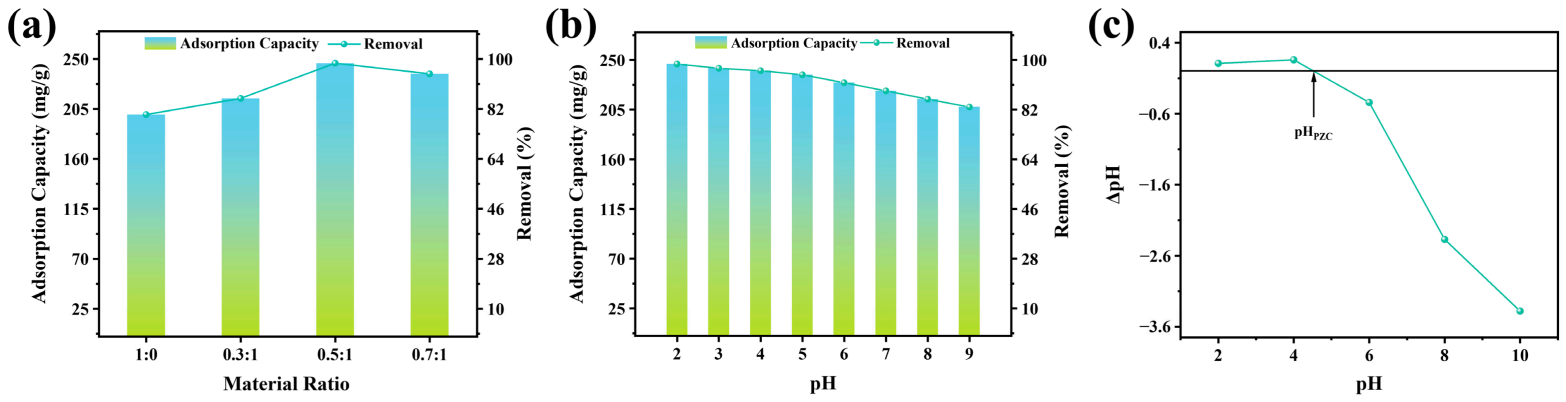
(**a**) The influence of raw material ratio on adsorption, (**b**) Impact of pH on adsorption, (**c**) pH_PZC_ measurement for MIL-100(Fe)@KTBC.

The effect of the raw material ratio on the adsorption performance of MIL-100(Fe)@KTBC toward XO is illustrated in Figure 6a. The results showed that the adsorption capacity of pristine MIL-100(Fe) was 199.90 mg/g. With the increase in the MIL-100(Fe) loading ratio, the adsorption capacity of the composite material was observed to first increase and then slightly decrease. When the mass ratio was 0.3, the adsorption capacity of MIL-100(Fe)_0.3_@KTBC was increased to 214.45 mg/g; as the ratio was raised to 0.5, the adsorption capacity reached 246.12 mg/g. Upon a further increase to 0.7, the adsorption capacity was decreased to 236.55 mg/g. It was found that an appropriate loading could increase the number of active sites and enhance the adsorption, whereas excessive loading resulted in agglomeration and pore blockage, by which the effective surface area and accessible active sites were reduced. This was consistent with the SEM observations: particle agglomeration was induced by excess MIL-100(Fe), which in turn resulted in a loss of active sites. Owing to the synergistic effect, MIL-100(Fe)_0.5_@KTBC was endowed with abundant active sites and a favorable pore structure; thereby, the highest adsorption capacity and removal efficiency were achieved, and it was identified as the optimal adsorbent in this series.

#### 2.2.2. Effect of Initial Solution pH on Adsorption Performance

The trends of the adsorption capacity and removal efficiency of XO by MIL-100(Fe)@KTBC as a function of solution pH are displayed in Figure 6b. It was indicated that both gradually decreased with increasing pH. As shown in Figure 6c, the point of zero charge (pH_PZC_) of MIL-100(Fe)@KTBC was determined to be 4.53. When pH was lower than pH_PZC_, the material surface was positively charged, and XO was predominantly present in its anionic form under acidic conditions. The electrostatic attraction between them promoted the adsorption process, through which relatively high adsorption capacity and removal efficiency were maintained [47]. As the pH was increased, the positive charge density on the material surface was decreased, the deprotonation of XO was enhanced, and the electrostatic repulsion was gradually intensified, leading to a decline in adsorption performance. Furthermore, under strongly alkaline conditions, OH^−^ ions were found to compete with XO anions for the active sites on the material surface, by which the adsorption was further inhibited [40]. Considering the adsorption performance and practical application requirements, it is suggested that the solution pH be adjusted to 4.0, whereby a relatively high adsorption performance can be maintained while the risks of equipment corrosion and environmental hazards caused by excessive acid addition are reduced.

#### 2.2.3. Effect of MIL-100(Fe)@KTBC Dosage on Adsorption Performance

**Figure 7:**
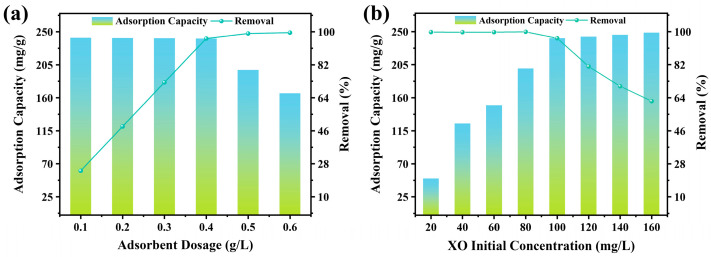
(**a**) Impact of adsorbent dosage on adsorption, (**b**) Impact of initial XO concentration on adsorption.

The adsorbent dosage is considered an important factor influencing the adsorption process, as it determines not only the number of available adsorption sites but also the mass transfer efficiency. The effect of MIL-100(Fe)@KTBC dosage on the adsorption performance of XO is presented in Figure 7a. When the dosage was increased from 0.1 g/L to 0.4 g/L, the adsorption capacity was maintained at approximately 240 mg/g, indicating that sufficient active sites were provided by the adsorbent within this range and XO molecules were effectively bound. Meanwhile, the removal efficiency was enhanced from 24.18% to 96.34%, which was mainly attributed to the increased number of active sites resulting from the higher adsorbent dosage, by which the contact opportunities between XO molecules and the adsorbent were improved. As the dosage was further increased to 0.6 g/L, a significant decrease in adsorption capacity was observed, whereas the removal efficiency tended to stabilize and was maintained at approximately 99%, suggesting that the system was approaching adsorption equilibrium. The decline in adsorption capacity was primarily caused by the “site-dilution effect” under a constant total XO concentration, i.e., the amount of XO loaded per unit mass of the adsorbent was reduced. By comprehensively considering both adsorption capacity and removal efficiency, 0.4 g/L was determined as the optimal dosage, at which a favorable balance between removal performance and material utilization efficiency was achieved.

#### 2.2.4. Effect of Initial XO Concentration on Adsorption Performance

The effect of the initial XO concentration on the adsorption performance of MIL-100(Fe)@KTBC is shown in Figure 7b. As the initial XO concentration was increased, the adsorption capacity gradually rose and then leveled off, whereas the removal efficiency shifted from being relatively stable in the initial stage to a gradual decline. When the XO concentration was raised from 20 to 100 mg/L, the adsorption capacity was enhanced from 49.87 to 240.94 mg/g, which was primarily attributed to the strengthened mass transfer driving force at higher concentrations, promoting sufficient contact between XO molecules and the active sites on the adsorbent surface. At initial concentrations below 100 mg/L, the XO removal efficiency was maintained above 99.5%, indicating that the adsorption sites were abundant and XO could be efficiently removed from the solution. After the concentration was further increased, the active sites became gradually occupied and approached saturation [46], resulting in a marked decrease in removal efficiency, which dropped to 62.13% at 160 mg/L. These results demonstrate that MIL-100(Fe)@KTBC exhibits excellent removal efficiency in the treatment of low-concentration XO wastewater and possesses a high adsorption capacity potential under high-concentration conditions, thus providing a basis for the optimization of practical application parameters.

#### 2.2.5. Effect of Coexisting Ions on Adsorption Performance

**Figure 8:**
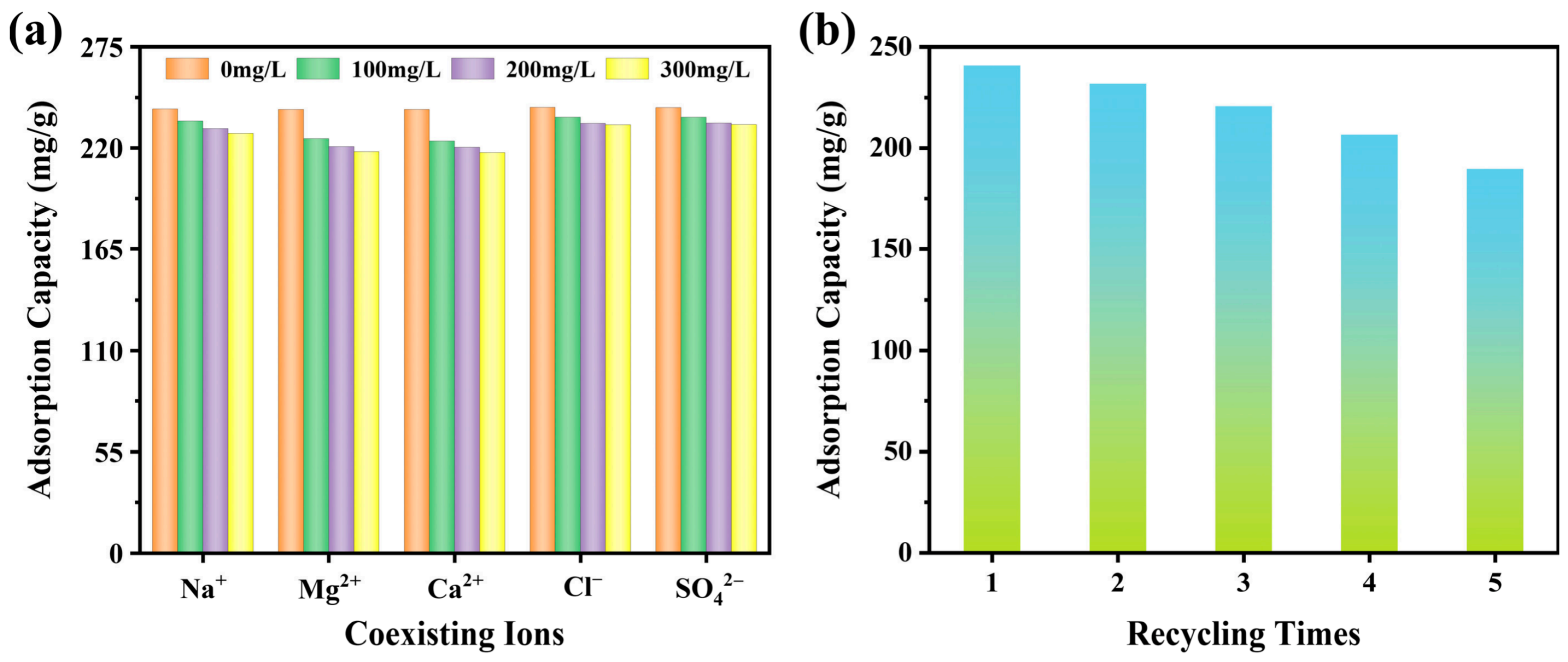
(**a**) Effect of coexisting ions on adsorption performance, (**b**) Cyclic regeneration stability of the adsorbent.

The effects of coexisting anions and cations on the adsorption performance of MIL-100(Fe)@KTBC toward XO are presented in Figure 8a. Overall, the presence of cations such as Na^+^, Ca^2+^, and Mg^2+^, as well as anions such as Cl^−^ and SO_4_^2−^, did not significantly inhibit the adsorption performance of the adsorbent, indicating that MIL-100(Fe)@KTBC possesses favorable resistance to ionic interference.

In the cationic systems, the adsorption capacity was only slightly decreased as the ion concentration was increased. Specifically, the effect of Na^+^ on the adsorption performance was relatively weak, whereas the inhibitory effects exerted by Mg^2+^ and Ca^2+^ were slightly stronger, which may be attributed to the higher charge density of divalent cations. These divalent cations can weaken the electrostatic interactions between the adsorbent and XO^n−^ by occupying the active sites on the adsorbent surface, enhancing the charge screening effect, and compressing the electrical double layer. Meanwhile, higher ionic strength may promote the aggregation of XO molecules, thereby hindering their diffusion and adsorption. In the anionic systems, no significant fluctuations in adsorption capacity were observed upon the addition of Cl^−^ and SO_4_^2−^, suggesting that their competitive effects are limited. Anions may compete with XO^n−^ for positively charged sites on the adsorbent surface and generate a certain degree of electrostatic repulsion.

The competitive adsorption of XO on MIL-100(Fe)@KTBC was examined with four coexisting dyes at a 1:1 ratio, as shown in Figure S2. Anionic methyl orange (MO) and congo red (CR) significantly reduced XO adsorption by competing for the same sites via electrostatic and π–π interactions. MO, due to its smaller size, preferentially occupied micropores; CR’s multiple sulfonate groups strengthened electrostatic competition. Cationic rhodamine B (RB) and methylene blue (MB) had a weaker effect, likely forming a ternary ‘adsorbent–cationic dye–XO’ structure through electrostatic attraction and π–π interactions, modulating surface charge and sustaining XO enrichment. This indicates MIL-100(Fe)@KTBC possesses anti-interference ability and selective adsorption potential in multi-component printing and dyeing wastewater.

#### 2.2.6. Cyclic Reuse Performance of the MIL-100(Fe)@KTBC Adsorbent

The cyclic adsorption stability is considered a crucial indicator for evaluating the practical applicability and long-term efficacy of MIL-100(Fe)@KTBC. In this study, the material was regenerated with 0.1 mol/L NaOH solution after each adsorption cycle. As shown in Figure 8b, a gradual decrease in the adsorption capacity of MIL-100(Fe)@KTBC for XO was observed with increasing cycle number. After five cycles, the adsorption capacity was reduced to 189.74 mg/g, and 78.82% of the initial capacity was retained, indicating that favorable regeneration performance and reusability were exhibited by the composite. The decline in adsorption capacity can be attributed to two factors: first, during repeated adsorption–desorption processes, some XO molecules or intermediate products might be retained within the pores, occupying effective adsorption sites; second, slight structural damage or loss of active sites might be induced by mechanical stirring, centrifugation, and other operations. In addition, because regeneration was conducted under strongly alkaline conditions, the possibility of partial hydrolysis of the Fe–carboxylate coordination bonds in MIL-100(Fe) and the subsequent release of Fe species cannot be completely excluded. If iron leaching occurs and accumulates over repeated regeneration cycles, it may reduce the number of Fe-containing active sites and weaken the structural integrity of the MIL-100(Fe) framework. It was demonstrated that MIL-100(Fe) and its decomposition products, Fe^3+^ ions and trimesic acid, were non-toxic and posed a low environmental risk, and that the associated risk could be relatively controlled through enhanced monitoring. Overall, a relatively high adsorption capacity was maintained by MIL-100(Fe)@KTBC after multiple cycles, demonstrating its potential application as an efficient and regenerable adsorbent for XO-containing wastewater.

To evaluate the relative performance and potential applicability of MIL-100(Fe)@KTBC, its adsorption capacity and preparation characteristics were compared with those of representative adsorbents previously reported for XO removal, as summarized in Table S1. These results demonstrate the considerable advantage of MIL-100(Fe)@KTBC in terms of adsorbent utilization efficiency. In this study, tomato-derived biochar was employed as a low-cost support and combined with an iron-based MOF; a significantly higher adsorption capacity for XO was simultaneously achieved.

### 2.3. Adsorption Kinetics

**Figure 9:**
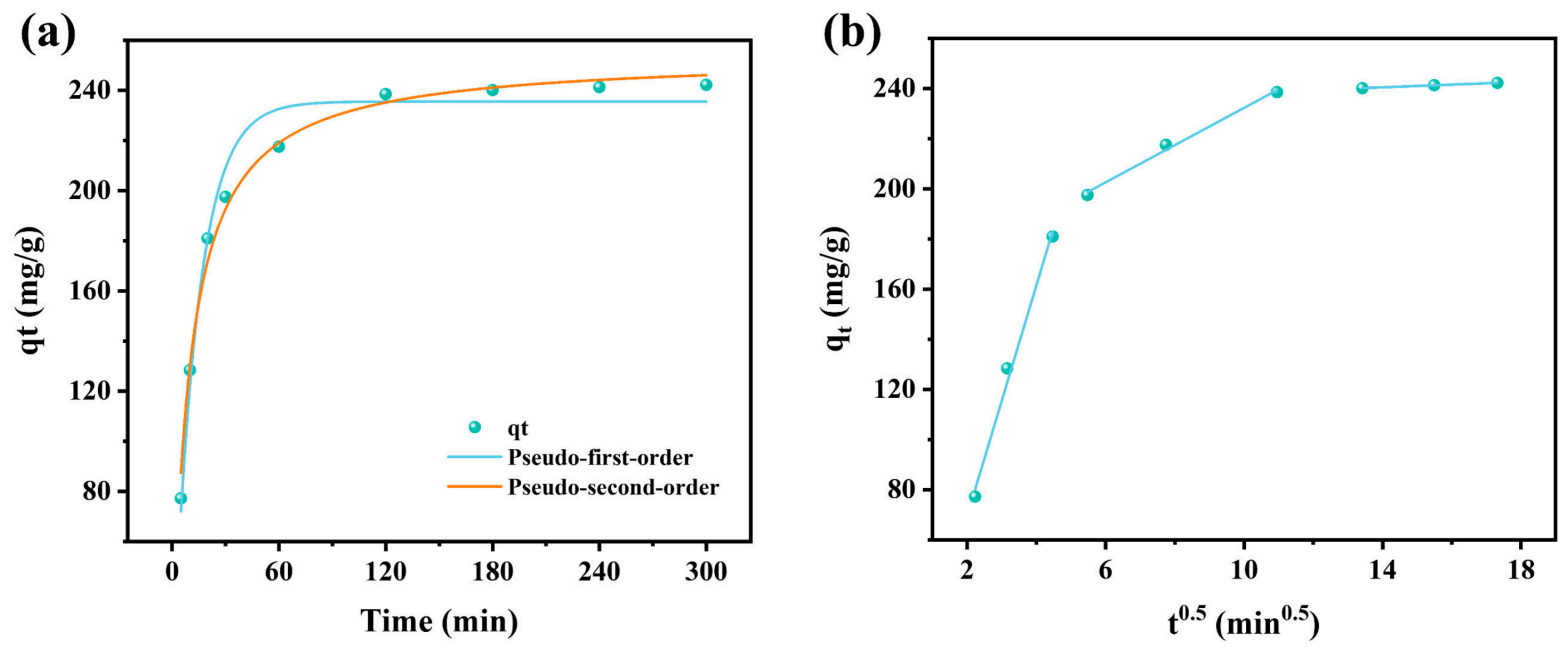
Adsorption kinetics of XO on MIL-100(Fe)_0.5_@KTBC: (**a**) pseudo-first-order/pseudo-second-order model fitting, (**b**) intraparticle diffusion model analysis.

To gain an in-depth understanding of the adsorption kinetic behavior of XO onto MIL-100(Fe)_0.5_@KTBC, the experimental data were fitted with the pseudo-first-order kinetic model (Equation (S3)), the pseudo-second-order kinetic model (Equation (S4)) [40], and the intra-particle diffusion model (Equation (S5)) [48], respectively. By comparing the fitting performance of the different models, the rate-controlling step and the underlying mechanism of the adsorption process can be further elucidated. The corresponding fitting curves are presented in Figure 9a,b, and the related kinetic parameters are summarized in Table 2. The equations of these kinetic models are given as follows:

As can be seen from Figure 9a, the variation in adsorption capacity with time was well described by both the pseudo-first-order and pseudo-second-order kinetic models. Specifically, the adsorption capacity increased rapidly at the initial stage, after which the rate of increase gradually declined and approached equilibrium, indicating that a high initial adsorption rate was attained and the system subsequently stabilized as the adsorption sites were progressively occupied. As shown in Table 2, the coefficient of determination (R^2^) of the pseudo-second-order model was higher than that of the pseudo-first-order model, and the calculated equilibrium adsorption capacity (q_e,cal_ = 250.55 mg/g) was closer to the experimental value, suggesting that the adsorption of XO onto MIL-100(Fe)_0.5_@KTBC was better described by the pseudo-second-order kinetic model. This indicates that the process was mainly governed by chemisorption.

To further elucidate the rate-controlling steps of the adsorption process, the intraparticle diffusion model was employed to perform piecewise linear fitting of the kinetic data, and the results are presented in Figure 9b. The adsorption process could be divided into three stages. Stage I was a rapid adsorption stage, with ki = 46.0179 mg/(g·min^0.5^) and C = 22.6838 mg/g, indicating that XO molecules were rapidly transferred from the bulk solution to the external surface of the adsorbent and that external diffusion exerted a significant influence on the adsorption rate. Stage II was a slow adsorption stage, in which a decrease in ki to 7.4296 mg/(g·min^0.5^) and an increase in C to 157.9853 mg/g were observed, corresponding to the gradual diffusion of XO molecules into the interior pores of the adsorbent, with intraparticle diffusion becoming the dominant rate-controlling step. Stage III was the equilibrium stage, in which ki further decreased to 0.5367 mg/(g·min^0.5^) and C reached 232.9655 mg/g, indicating that the adsorption sites tended to become saturated and that a dynamic equilibrium between adsorption and desorption was gradually attained. The R^2^ values for the fitting of each stage were all greater than 0.99, confirming that the kinetic characteristics at different stages could be effectively described by the intraparticle diffusion model. In addition, none of the fitted lines passed through the origin, suggesting that intraparticle diffusion was not the sole rate-controlling step and that a significant contribution to the adsorption process was also made by external diffusion [45]. In summary, the adsorption kinetics of XO onto MIL-100(Fe)_0.5_@KTBC were better described by the pseudo-second-order kinetic model, and the adsorption process was jointly controlled by external diffusion and intraparticle diffusion, with chemisorption playing the dominant role.

### 2.4. Adsorption Isotherms

**Figure 10:**
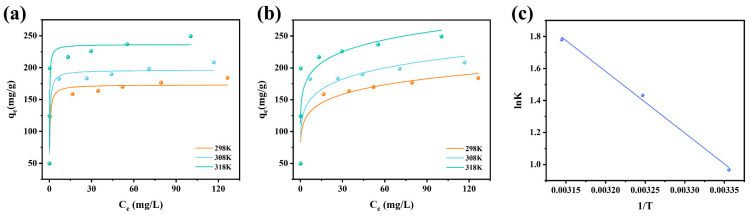
Adsorption isotherm fitting and thermodynamic analysis of XO on MIL-100(Fe)_0.5_@KTBC: (**a**) Langmuir model, (**b**) Freundlich model, (**c**) Van’t Hoff curve.

To investigate the adsorption behavior of XO onto the MIL-100(Fe)_0.5_@KTBC composite, the Langmuir and Freundlich isotherm models (Equations (S6) and (S7)) were fitted to the adsorption data obtained at different temperatures [45]. The fitted curves are presented in Figure 10a and Figure 10b, respectively, and the corresponding isotherm parameters are listed in Table 3. The equations of the isotherm models are given as follows.

As observed in Figure 10a, good agreement between the Langmuir model fitting curves and the experimental data is obtained, suggesting that the adsorption of XO onto MIL-100(Fe)_0.5_@KTBC may be dominated by monolayer adsorption and that the adsorption sites possess relatively homogeneous affinity. The equilibrium adsorption capacity (q_e_) is markedly increased with rising temperature, indicating that the adsorption process is endothermic in nature. Additionally, all determination coefficients (R^2^) of the Langmuir model are found to exceed 0.90, further confirming its satisfactory applicability to the present system. In contrast, as presented in Figure 10b, although the Freundlich model is able to reflect the overall trend of adsorption capacity with concentration, the obtained R^2^ values are generally lower than those of the Langmuir model, indicating a relatively weaker fitting performance. In the Freundlich model, the K_F_ values are increased with increasing temperature, suggesting that the adsorption capacity is gradually enhanced; all n values are greater than 1, implying that the adsorption process is favorable. Overall, the Langmuir model is demonstrated to be more appropriate for describing the adsorption behavior of XO on MIL-100(Fe)_0.5_@KTBC, revealing that monolayer coverage is formed predominantly on homogeneous surface active sites [45]. Furthermore, the endothermic nature of the process is further evidenced by the increased adsorption capacity with temperature, which may be attributed to chemisorption or strong physical interactions. These results can serve as a theoretical basis for the optimization of adsorption conditions and practical applications.

### 2.5. Adsorption Thermodynamics

To further investigate the thermodynamic characteristics of the adsorption process, the equilibrium constant K_L_ obtained from Langmuir model fitting was used to construct a plot of lnK_L_ versus 1/T (Figure 10c) based on the Van’t Hoff Equation (S8), and a linear fitting was performed [40]. The results indicated that the adsorption process conformed to the Van’t Hoff model. The values of ΔH and ΔS were calculated from the slope and intercept of the fitted line, respectively. Subsequently, ΔG at different temperatures was determined using the Gibbs Equation (S9), and the obtained thermodynamic parameters are listed in Table 4.

The calculated results revealed that ΔH > 0, confirming that the adsorption of XO onto MIL-100(Fe)_0.5_@KTBC is an endothermic process and that the adsorption is promoted by elevated temperature, which is consistent with the observed increase in qm with temperature in the isotherms. ΔS > 0 indicates that the disorder of the system is increased during the adsorption, which may be attributed to the release of bound water molecules from the solvation layer of XO as these molecules are transferred from the liquid phase to the adsorbent surface. Over the experimental temperature range, the ΔG values were all negative, and their absolute values increased with rising temperature, demonstrating that the adsorption process is spontaneous and that the spontaneity is strengthened under higher-temperature conditions. In summary, the adsorption of XO onto MIL-100(Fe)_0.5_@KTBC is a spontaneous, endothermic, and entropy-driven process, and elevated temperature is more favorable for the adsorption [45].

### 2.6. Analysis of Adsorption Mechanism

Based on the integrated analysis of adsorption kinetics, isotherms and thermodynamics, it was established that the adsorption of XO onto MIL-100(Fe)@KTBC was primarily governed by chemisorption, exhibited homogeneous monolayer adsorption characteristics, and the process occurred spontaneously. However, it could not be directly verified from the kinetic modeling that chemisorption was the sole rate-controlling step; instead, the adsorption rate was found to be closely correlated with the accessibility and interactions of surface active sites. The intraparticle diffusion model displayed distinct multi-stage features. Initially, XO was rapidly transported and enriched on the external surface of the adsorbent. Subsequently, XO molecules gradually diffused into the interior pores, and the adsorption rate decreased as effective active sites were consumed and mass transfer resistance was intensified. Finally, adsorption equilibrium was attained. These results suggested that the XO adsorption process was co-regulated by both surface chemical interactions and intrapore diffusion. To further elucidate the adsorption mechanism, the surface morphology, elemental composition and distribution, and changes in functional groups and chemical states of the materials before and after adsorption were systematically characterized by SEM-EDS, FTIR, and XPS.

**Figure 11:**
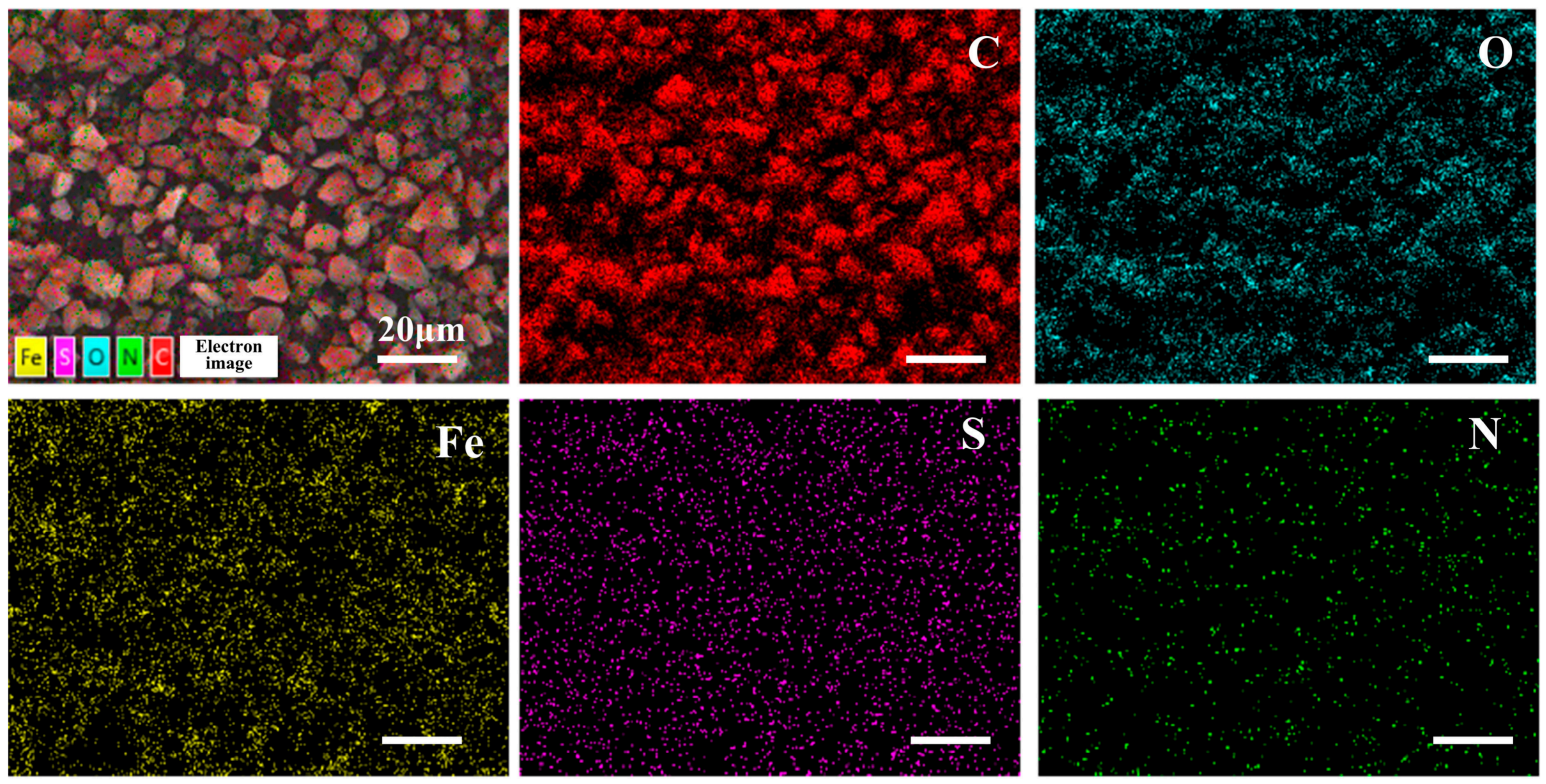
Elemental mapping of C, O, Fe, S, and N in MIL-100(Fe)_0.5_@KTBC after XO adsorption.

To investigate the adsorption mechanism of XO onto MIL-100(Fe)_0.5_@KTBC, SEM-EDS elemental mapping analysis was performed on the post-adsorption material (Figure 11). The EDS mapping results showed that C, O, Fe, S, and N were uniformly and continuously distributed across the sample area, without obvious local enrichment or deficiency. Among them, the signals of C and O were strong and widely distributed, which is consistent with the carbon-dominated nature of the KTBC carbonaceous carrier and with the characteristic abundance of C, O, and Fe in the organic ligands and metal nodes of MIL-100(Fe) [47]. The distribution of Fe was highly overlapped with those of C and O, indicating that MIL-100(Fe) nanoparticles had been in situ grown on the surface and within the pores of KTBC, forming a stable composite structure that provided a structural basis for efficient XO adsorption. After adsorption, distinct characteristic signals of S and N were detected in the sample, and their distributions were essentially consistent with those of Fe, C, and O, demonstrating that XO molecules bearing sulfonic acid and imino groups had been successfully loaded onto the surface and into the pores of the composite. The uniform elemental distribution further indicated that XO adsorption was relatively homogeneous, without obvious local agglomeration, which was beneficial to maintaining the stability of the adsorption performance of the material.

**Figure 12:**
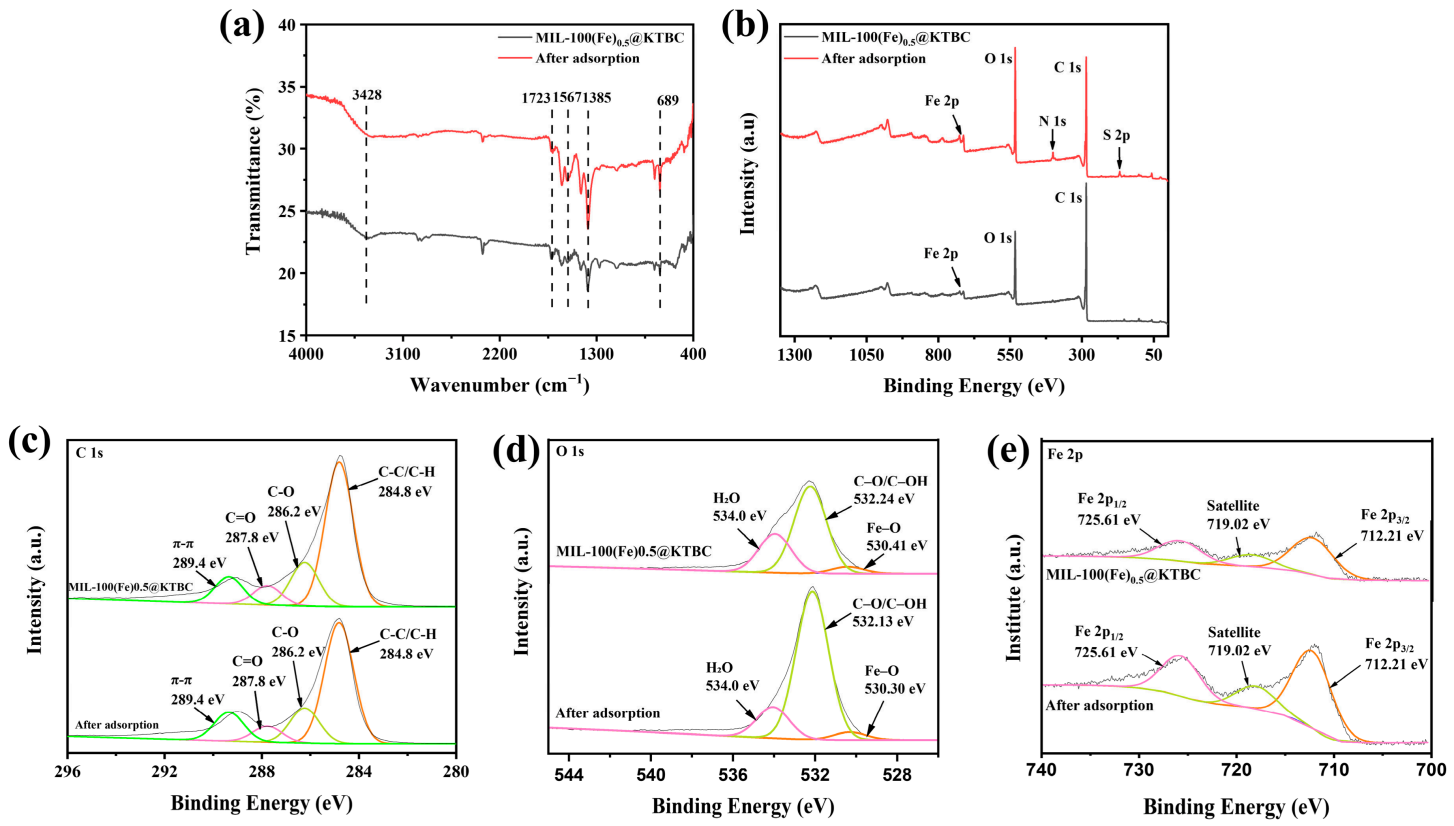
Characterization comparison of MIL-100(Fe)_0.5_@KTBC before and after XO adsorption: (**a**) FTIR spectra, (**b**–**e**) XPS spectra.

As revealed by the FTIR spectra of MIL-100(Fe)_0.5_@KTBC before and after XO adsorption in Figure 12a, the O–H stretching vibration peak at 3428 cm^−1^ was broadened, and its intensity was reduced, indicating that hydrogen bonds were formed between the phenolic hydroxyl/carboxyl groups of XO and the hydroxyl groups on the adsorbent surface, by which the O–H vibration was weakened [44]. The C=O stretching vibration peak at 1627 cm^−1^ was enhanced, which could be attributed to competitive coordination between the BTC carboxyl groups of MIL-100 and the carboxyl groups of XO, or to the coordination of deprotonated XO carboxyl groups with Fe^3+^ [37]. The characteristic vibration peaks of the benzene ring and azo bond of XO at 1567 cm^−1^ were enhanced, suggesting the existence of π–π stacking and conjugation interactions. The symmetric stretching vibration peak of carboxyl groups at 1385 cm^−1^ was enhanced, which was associated with the superposition of vibrations from the phenolic hydroxyl and sulfonic acid groups of XO and the carboxyl groups of the adsorbent [45]. The change in the Fe–O vibration at 689 cm^−1^ further indicated that Fe^3+^ was coordinated with the carboxyl (–COO^−^), phenolic hydroxyl (–OH), and sulfonic acid (–SO_3_^−^) groups of XO [49].

As shown in the XPS survey spectrum in Figure 12b, after the adsorption of XO onto MIL-100(Fe)_0.5_@KTBC, new characteristic peaks of S 2p and N 1s were observed, which are assigned to the sulfonic acid group and imino group of XO molecules, respectively, indicating that XO was successfully adsorbed on the composite surface.

In the C 1s spectrum (Figure 12c), the C–C/C–H peak at 284.8 eV is attributed to aromatic rings and aliphatic hydrocarbon structures; after adsorption, its peak area percentage was significantly reduced, suggesting that the active sites on the material surface were covered by XO molecules [37]. The C–O peak at 286.2 eV is assigned to hydroxyl and ether bonds; its decreased proportion after adsorption indicates that the hydroxyl groups may be involved in hydrogen bonding or complexation with XO [35]. The proportion of the C=O peak at 287.7 eV was slightly lowered, which might be related to the change in surface microenvironment caused by XO loading. The π–π* satellite peak at 289.5 eV was remarkably enhanced, indicating that π–π stacking interactions were formed between the aromatic rings of XO and the benzene rings in MIL-100(Fe)_0.5_@KTBC [43].

From the O 1s spectrum in Figure 12d, the binding energy of the Fe–O bond was slightly shifted from 530.41 eV to 530.30 eV, and its peak area was decreased, suggesting that the Fe sites were coordinated with oxygen-containing groups of XO such as hydroxyl, carboxyl, and sulfonic acid groups, and that the electron cloud of the Fe–O bond was shifted toward the XO molecule [35]. Meanwhile, the binding energy of the C–O/C–OH peak was decreased from 532.24 eV to 532.13 eV, and its peak area was increased, indicating that hydrogen bonds were formed between the oxygen-containing functional groups of XO and the C–O groups on the composite surface, which promoted the enrichment of oxygen-containing species on the surface [37]. The peak area of the characteristic peak of adsorbed water (534.0 eV) was reduced, indicating that water molecules were displaced by XO molecules during the adsorption process and that the surface active sites were effectively utilized [49].

In the Fe 2p spectrum (Figure 12e), after adsorption, the Fe 2p3/2 and Fe 2p1/2 peaks were shifted from 712.46 and 725.97 eV to 712.21 and 725.61 eV, respectively, and the satellite peak was shifted from 718.50 eV to 719.02 eV, indicating that electron transfer occurred between the Fe sites and XO and that the Fe coordination environment was slightly reconstructed [37]. The peak area changed only slightly, suggesting that Fe^3+^ was generally stable and that the Fe^3+^ sites, as the key active centers, were involved in the XO adsorption process.

**Figure 13:**
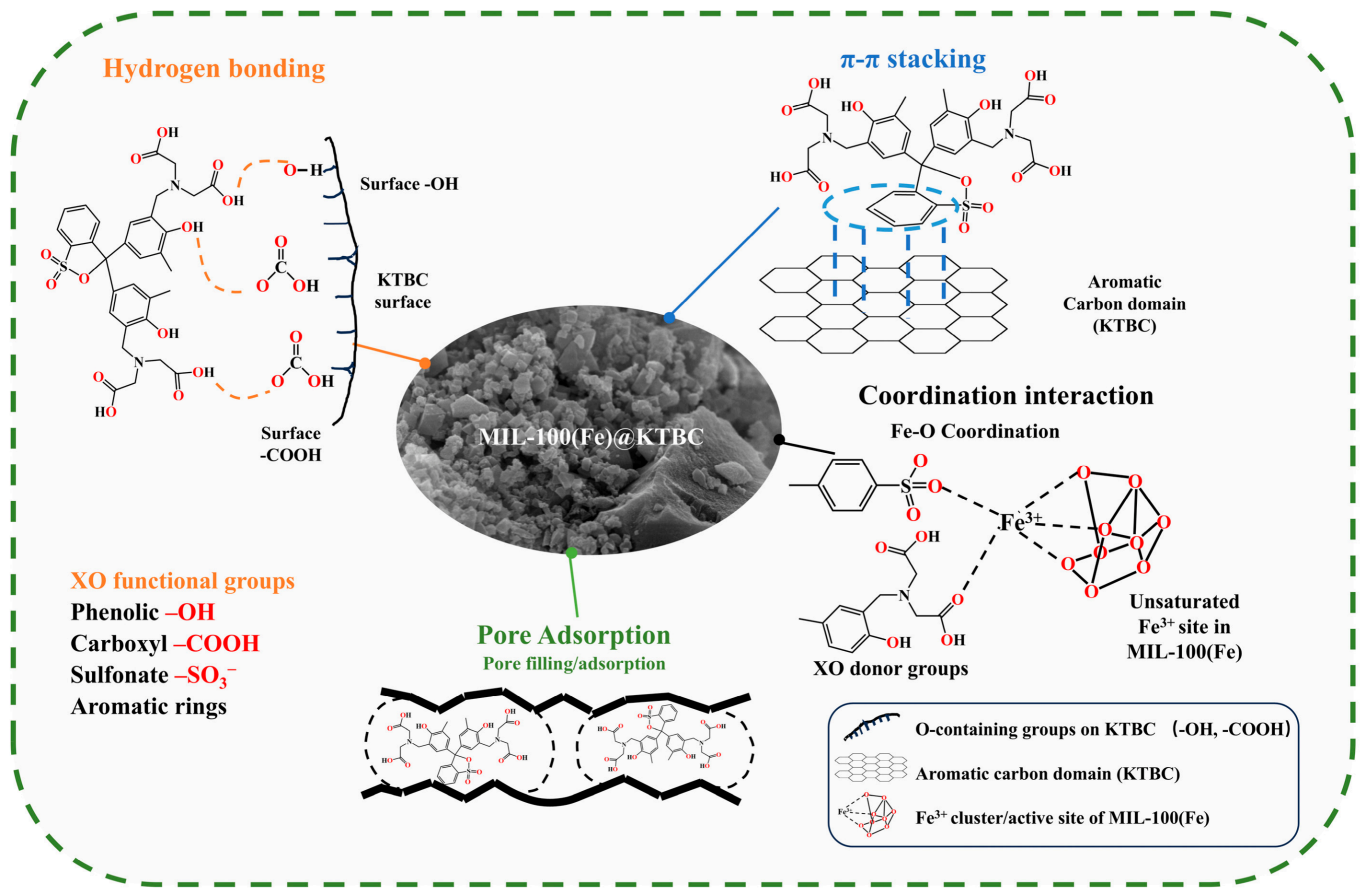
Mechanism of XO adsorption on MIL-100(Fe)@KTBC.

Based on the characterization results and experimental data, the adsorption mechanism of XO on MIL-100(Fe)@KTBC was reasonably elucidated in this work, as illustrated in Figure 13.

## 3. Materials and Methods

### 3.1. Reagents and Materials

Tomato straw was obtained from the Shouguang Vegetable Production Base. Ferric nitrate nonahydrate was purchased from Sinopharm Chemical Reagent Co., Ltd. (Shanghai, China). Benzene-1,3,5-tricarboxylic acid, also known as trimesic acid, 99%, was supplied by Beijing Innochem Technology Co., Ltd. (Beijing, China). Ethanol, sodium bicarbonate, and xylenol orange were purchased from Tianjin Kemiou Chemical Reagent Co., Ltd. (Tianjin, China). Potassium hydroxide was obtained from Yantai Yuandong Fine Chemicals Co., Ltd. (Yantai, China).

### 3.2. Preparation of MIL-100(Fe)@KTBC

**Figure 14:**
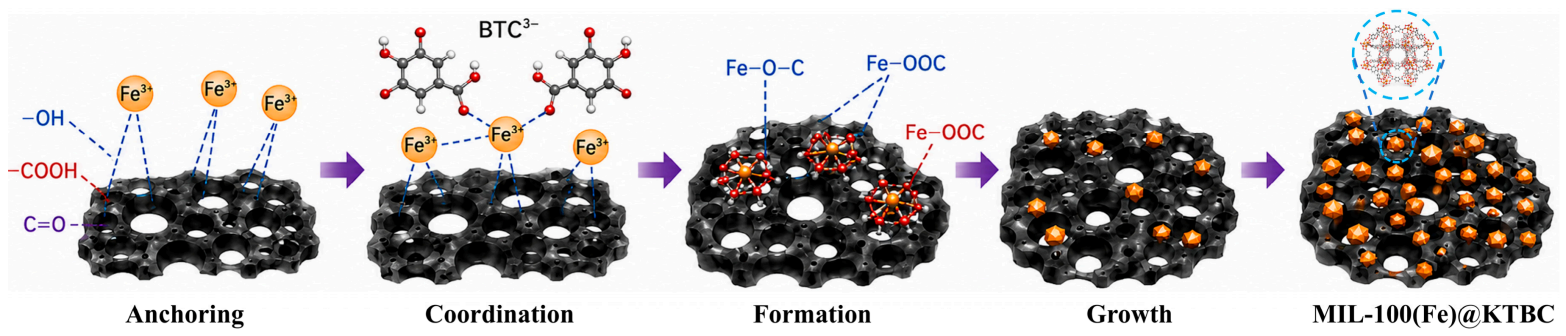
Schematic diagram of the preparation of MIL-100(Fe)@KTBC.

Tomato biochar was prepared according to the method reported by Zheng et al. [40], followed by high-temperature KOH activation to obtain KTBC with a high specific surface area. Briefly, 6.733 g of iron(III) nitrate nonahydrate and 2.345 g of trimesic acid were dissolved in 50 mL of 0.05 mol/L sodium bicarbonate solution. The mixture was ultrasonically dispersed for 15 min and then stirred for 30 min. Subsequently, a certain amount of KTBC was added, followed by further ultrasonication for 15 min and stirring for 1 h. The resulting suspension was transferred into a Teflon-lined stainless-steel autoclave and subjected to hydrothermal treatment at 160 °C for 12 h. After cooling, the solid product was collected by centrifugation and sequentially washed with deionized water at 80 °C under stirring and anhydrous ethanol under reflux, each for 3 h, followed by centrifugation. Finally, the product was vacuum-dried at 90 °C for 12 h to obtain MIL-100(Fe)_x_@KTBC composites, where x represents the mass ratio of MIL-100(Fe) to KTBC (x = 0.3, 0.5, and 0.7). A schematic illustration of the preparation process of MIL-100(Fe)@KTBC is shown in Figure 14.

### 3.3. Characterization

In this study, multiple characterization techniques were employed to systematically investigate the morphology, structure, and physicochemical properties of the MIL-100(Fe)@TBC composite. The microstructure and morphology of the materials were observed by scanning electron microscopy (SEM; Hitachi SU8600, Hitachi, Tokyo, Japan) and transmission electron microscopy (TEM; Talos F200i, Thermo Fisher Scientific, Waltham, MA, USA). The crystal structure was analyzed by X-ray diffraction (XRD; Rigaku, Tokyo, Japan) over a scanning range of 5–60° at a scanning rate of 5°/min. Raman spectroscopy (HORIBA XploRA PLUS, HORIBA, Kyoto, Japan, 532 nm) was used to characterize the ordered and disordered structures of graphitic-like carbon. Fourier-transform infrared spectroscopy (FTIR; Beifen-Ruili, Beijing, China) was performed to analyze the functional groups within the scanning range of 4400–400 cm^−1^. The specific surface area and pore size distribution were determined based on the Brunauer–Emmett–Teller (BET) method using a Micromeritics ASAP 2460 analyzer (Micromeritics, Norcross, GA, USA). Thermogravimetric stability was evaluated by simultaneous thermal analysis (STA; Hitachi STA200RV, Hitachi, Tokyo, Japan) under a nitrogen atmosphere from room temperature to 800 °C at a heating rate of 10 °C/min.

### 3.4. Adsorption Experiments

To evaluate the adsorption performance of the MIL-100(Fe)@KTBC composite toward XO, a series of batch adsorption experiments was conducted. Briefly, 0.01–0.06 g of adsorbent was added to 100 mL of XO solution with initial concentrations ranging from 20 to 200 mg/L. The experiments were performed under different pH values and contact times. After adsorption equilibrium was reached, solid–liquid separation was carried out using a 0.22 μm mixed cellulose ester microporous membrane filter. The residual XO concentration in the filtrate was then determined by UV–vis spectrophotometry at 436 nm. Both the removal efficiency and adsorption capacity were calculated according to Equations (S1) and (S2). The optimal adsorbent sample was subsequently selected, and adsorption kinetic and thermodynamic analyses were further performed to elucidate the adsorption mechanism and process characteristics.

## 4. Conclusions

Using KTBC as the support, Fe^3+^ was coordinated and anchored by the oxygen-containing functional groups on its surface, thereby inducing the uniform in situ nucleation and crystallization of MIL-100(Fe). As a result, MIL-100(Fe)@KTBC composites were successfully prepared, and the confined growth and firm immobilization of the MOF were achieved. The characterization results demonstrated that the porous carbon framework of KTBC effectively suppressed the aggregation of MIL-100(Fe) particles and improved the pore structure, thermal stability, and structural stability of the composites. When the mass ratio of MIL-100(Fe) to KTBC was 0.5, the composite exhibited both a high exposure degree of active sites and a favorable pore structure. Under the conditions of pH 4.0 and an adsorbent dosage of 0.4 g/L, the highest adsorption capacity toward XO was obtained, reaching 246.12 mg/g.

The adsorption kinetic results showed that the adsorption of XO onto MIL-100(Fe)_0.5_@KTBC was better fitted by the pseudo-second-order kinetic model, indicating that chemisorption played a dominant role in the adsorption process. The intraparticle diffusion fitting results revealed that the adsorption process consisted of three stages: rapid adsorption on the external surface, diffusion within the pores, and adsorption equilibrium. Both external diffusion and intraparticle diffusion were found to jointly control the adsorption rate. The adsorption isotherm results indicated that the Langmuir model described the adsorption process more appropriately than the Freundlich model, suggesting that XO was mainly adsorbed as a monolayer on relatively homogeneous active sites.

Thermodynamic analysis showed that ΔG was negative, whereas both ΔH and ΔS were positive, confirming that the adsorption process was spontaneous, endothermic, and entropy-driven. Thus, an increase in temperature was favorable for adsorption. The coexistence of anions and cations did not cause a significant decrease in the adsorption capacity of the composite toward XO, demonstrating its excellent anti-interference capability. After five adsorption–desorption cycles, the composite still maintained a high adsorption capacity for XO. Moreover, the regenerated material exhibited excellent structural and chemical stability, indicating good recyclability and practical application potential. Mechanistic investigations revealed that coordination interactions, π–π stacking interactions, hydrogen bonding, and pore-filling effects were the primary driving forces for the adsorption process.

Despite these promising results, the present study was primarily conducted using simulated XO-containing solutions under controlled laboratory conditions. The adsorption performance of MIL-100(Fe)_0.5_@KTBC may be affected by the complex composition of real wastewater, including dissolved organic matter, suspended solids, variable pH, high ionic strength, and competing contaminants. Therefore, further studies using actual industrial wastewater are required to evaluate the selectivity, stability, regeneration efficiency, and long-term adsorption performance of the composite under realistic conditions. In addition, pilot-scale continuous-flow experiments should be conducted to assess its dynamic adsorption behavior, operational stability, treatment efficiency, and economic feasibility before large-scale practical application.

## Figures and Tables

**Table 1:** Specific surface area and pore properties data of MIL-100(Fe)@KTBC.

Sample	Specific Surface Area (m^2^/g)	Pore Diameter (nm)	Pore Volume (cm^3^/g)
MIL-100(Fe)	735.63	7.28	0.228
MIL-100(Fe)0.3@KTBC	1566.40	3.33	0.673
MIL-100(Fe)0.5@KTBC	1726.82	4.59	0.628
MIL-100(Fe)0.7@KTBC	1481.77	6.22	0.733

**Table 2:** Kinetic Parameters for XO Adsorption onto MIL-100(Fe)_0.5_@KTBC.

Models	Parameters	Values
Pseudo-first-order	q_e,cal_ (mg/g)	239.46
	K_1_ (1/min)	0.0528
	R^2^	0.9988
Pseudo-second-order	K_2_ [g/(mg·min)]	4.866 × 10^−4^
	q_e,cal_ (mg/g)	250.55
	R^2^	0.9998
Intraparticle diffusion	Phase I	
	k_i_(mg/g·min^0.5^)	46.0179
	C(mg/g)	22.6838
	R^2^	0.9925
	Phase II	
	k_i_(mg/g·min^0.5^)	7.4296
	C(mg/g)	157.9853
	R^2^	0.9928
	Phase III	
	k_i_(mg/g·min^0.5^)	0.5367
	C(mg/g)	232.9655
	R^2^	0.9979

**Table 3:** Isotherm parameters for XO adsorption onto MIL-100(Fe)_0.5_@KTBC composite.

T (K)	Langmuir Model			Freundlich Model		
	q_m_ (mg/g)	K_L_ (L/mg)	R^2^	K_F_ (mg/g)	n	R^2^
298	173.23	2.63	0.9307	101.62	7.66	0.8318
308	196.23	2.78	0.9555	115.72	7.50	0.8216
318	236.39	5.93	0.9041	151.74	8.62	0.7308

**Table 4:** Thermodynamic parameters for the adsorption of XO onto MIL-100(Fe)_0.5_@KTBC.

T (K)	lnK_L_	∆G (KJ·mol^−1^)	∆H (KJ·mol^−1^)	∆S (J/mol·K)
298	0.9670	−2.3958	32.0670	115.7708
308	1.4325	−3.6682		
318	1.7800	−4.7061		

## Data Availability

The original contributions presented in the study are included in the article; further inquiries can be directed to the corresponding authors.

## Supplementary Materials

The following supporting information can be downloaded at: https://www.mdpi.com/article/10.3390/molecules31152604/s1. Figure S1: SEM images of MIL-100(Fe); Figure S2: Interference of coexisting dyes on adsorption performance; Table S1: Comparison of the XO adsorption capacity of various adsorbents. Refs. [50,51,52,53,54,55,56] are cited in the Supplementary Materials.
