# Supplementary material for: In Situ-Grown MIL-100(Fe) for Interfacial Regulation of KTBC and Its Adsorption Performance and Mechanism for Xylenol Orange Removal
Source: Molecules. 2026 Jul 25;31(15):2604. doi: 10.3390/molecules31152604 (PMC13468508; doi:10.3390/molecules31152604)
Supplement: Supplementary file 1 [file molecules-31-02604-s001.zip › molecules-4427063-supplementary.pdf]

In Situ-Grown MIL-100(Fe) for Interfacial Regulation of KTBC and Its Adsorption Performance and Mechanism for Xylenol Orange Removal

Shirui Zheng[1], Jinting Jiang[2], Zhihao Fang[2], Fangfang Liu[2] and Yongwei Li[2,*]

[1] State Key Laboratory of Bio-Fibers and Eco-Textiles, College of Materials Science and Engineering, Shandong Collaborative Innovation Center of Marine Biobased Fibers and Ecological Textiles, Qingdao University, Qingdao 266071, China; threezheng@aliyun.com
[2] School of Chemical Engineering and Environment, Weifang University of Science and Technology, Weifang 262700, China; jinn0717@163.com (J.J.); wfsgfzh@163.com (Z.F.); liuff10507@wfust.edu.cn (F.L.)
* Correspondence: liyongwei@wfust.edu.cn

Calculation formula of adsorption performance

Equations (S1) and (S2) were used to calculate the XO removal efficiency and adsorption capacity of the adsorbent.

$$R=\frac{C_0-C_t}{C_0}\times 100\% \tag{S1}$$

$$q_t=\frac{C_0-C_t}{m}\times V \tag{S2}$$

In these expressions: C_0 and C_t represent the initial concentration and the concentration of XO at time t (mg/L), respectively; V is the volume (L) of XO solution; m signifies the mass (g) of MIL-100(Fe)@KTBC; and q_t denotes the adsorption capacity at time t (mg/g).

Kinetic Model

To explore the kinetic mechanism of the adsorption process, this study employed the pseudo-first-order kinetic model (Equation S3) and pseudo-second-order kinetic model (Equation S4), respectively, to characterize the potentially involved physical and chemical adsorption behaviors. In addition, to further elucidate the mass transfer characteristics of XO molecules within the MIL-100(Fe)@KTBC material, the intra-particle diffusion model (Equation S5) was employed to analyze the internal diffusion and any concomitant proton transfer. Subsequently, the experimental data were fitted nonlinearly using the kinetic Equations (S3), (S4), and (S5).

$$q_t=q_e(1-e^{-k_1 t}) \tag{S3}$$

$$q_t=\frac{q_e^2 k_2 t}{1+q_e k_2 t} \tag{S4}$$

$$q_t=k_i t^{0.5}+C \tag{S5}$$

where q_t and q_e represent the adsorption capacity at contact time t and at adsorption equilibrium, respectively (mg/g). k_1, k_2 and k_i refer to the rate constants of the pseudo-first-order kinetic model (1/min), the pseudo-second-order kinetic model (1/min) and the intraparticle diffusion adsorption model (mg/g·$min^{0.5}$), respectively; t refers to the adsorption time in the adsorption process (min), and C is the boundary layer effect constant in the intraparticle diffusion model (mg/g).

Isotherm Models

To evaluate the adsorption data, the Langmuir and Freundlich isotherm models were employed, each offering complementary insight into the fundamental adsorption behavior. The
